# Prognostic Observational Analysis of BMI, Leptin, and Adiponectin in Children With Acute Lymphocytic Leukemia Undergoing Remission-Induction Chemotherapy

**DOI:** 10.3389/fped.2022.797836

**Published:** 2022-03-21

**Authors:** Jing Sun, Ru Zhang, Jianjun Tang, Xuedong Wu, Lu Zhu, Haiying Huang, Huimin Chen, Minhua Xiao, Hongfeng Luo, Haiqing Zheng, Jiaqi Chen

**Affiliations:** ^1^Department of Clinical Nutrition, Guangzhou Women and Children's Medical Center, Guangzhou, China; ^2^Department of Hematology and Oncology, Guangzhou Women and Children's Medical Center, Guangzhou, China; ^3^Department of Anesthesiology, Nanfang Hospital, Southern Medical University, Guangzhou, China; ^4^Department of Pediatrics, Nanfang Hospital, Southern Medical University, Guangzhou, China; ^5^Institute of Pediatrics, Guangzhou Women and Children's Medical Center, Guangzhou, China

**Keywords:** childhood acute lymphoblastic leukemia, remission-induction chemotherapy, obesity, body mass index, minimal residual disease (MRD), adipocytokine

## Abstract

**Background:**

The survival rate of children and adolescents with acute lymphoblastic leukemia (ALL) has progressively improved. However, ALL survivors often have adverse effects after treatment, such as an increased risk of obesity. Obesity has been associated with reduced survival.

**Objective:**

We investigated the relationship between obesity, adipocytokine levels, and ALL short-term outcomes.

**Methods:**

Weight and height were measured, and body mass index (BMI) was calculated at patient diagnosis and discharge. Leptin and Adiponectin levels and Minimal Residual Disease (MRD) were measured before therapy, at days 19 of remission-induction therapy, and at the end of remission-induction therapy (days 46). The relationship between BMI, adipocytokine levels, and MRD was then determined.

**Results:**

Compared to the normal BMI group, children with an abnormal increase in BMI had an increase in MRD at day 19 and 46 (*P* = 0.04 and *P* = 0.008), and showed a positive correlation (*P* = 0.014). In addition, we found a positive correlation between weight, hip circumference at diagnosis and at day 19, and MRD at day 46. Both BMI and fat concentric distribution affected the outcome of ALL children. A higher BMI was also associated with a significant increase in Leptin levels at diagnosis. Leptin resistance should be considered in ALL children with high BMI.

**Conclusion:**

BMI affects the outcome of ALL patients. Early interventions such as regular weight, height monitoring, and dietary assessments should be preferably initiated during remission-induction chemotherapy.

## Introduction

The survival rate of children and adolescents with acute lymphoblastic leukemia (ALL) has improved to more than 90% in developed countries as a result of improved risk-directed treatment and supportive care (1–4). However, ALL survivors often show adverse effects after their treatment, such as an increased risk of obesity and short stature (5). In children with acute myeloid leukemia, obesity at diagnosis has been associated with worse outcomes (6, 7). These include reduced survival and increased drug toxicity (8). In the long-term, obesity could result in substantial physical and psychosocial morbidity, such as infections, hypertension, and hyperglycemia during therapy, as well as metabolic syndrome (MS) later in life (9). Hence, MS criteria, such as BP, weight, and serum triglyceride levels, should be measured at each patient visit (10).

Obesity/overweight, one of the major complications after ALL treatment, affects treatment efficacy and survival through multiple mechanisms (11). Leptin is a protein synthesized in the fatty tissues and is effective for the control of obesity. Monitoring leptin levels in acute lymphoblastic leukemia (ALL) survivors could be helpful in controlling obesity (12). Biomarkers such as homeostasis model of assessment for insulin resistance index (HOMA-IR), leptin, and leptin/adiponectin have been associated with components of MS and have been associated with a greater risk of MS among ALL children (13).

This study investigated the adverse effects of obesity in ALL children in two centers who were treated using the St. Jude total XV protocol (14). This was done during the remission-induction phase, which is the most important phase during the chemotherapy protocol. Body mass index (BMI), waist circumference, and hip circumference at diagnosis and at the end of remission-induction were measured. Leptin, Adiponectin levels, and Minimal Residual Disease (MRD) (15) were measured at day 0, 19, and 46 of remission-induction chemotherapy using marrow examinations.

## Materials and Methods

### Study Design and Patients

This study was a longitudinal observational study. Between March 2017 and March 2018, ALL patients who received treatment at the Department of Hematology and Oncology of Guangzhou Women and Children's Medical Center and Department of Pediatrics at Nanfang Hospital, Southern Medical University were enrolled to this study. Patient inclusion criteria were: (1) Between 1 and 10 years of age; (2) newly diagnosed with ALL (16, 17) and received remission-induction chemotherapy based on the St. Jude total XV protocol (14) (Table 1); (3) clear consciousness and the ability to communicate, or were accompanied by their parent(s) or guardian; and (4) had stable disease. Patient exclusion criteria were: (1) extreme malnutrition or critical illness; (2) congenital metabolic or genetic disorders, such as urea cycle disorder or Prader-Willi syndrome; (3) psychiatric disorders or organic encephalopathy; (4) did not receive a normal diet or oral nutrition or required fasting and parenteral nutrition due to the disease condition.

**Table 1:** Remission-induction chemotherapy.

		**Group**	**Dose**	**Route**	**Frequency**	**Time (days)**	**Note**
Window	Dex	L/I/H	6 mg/m^2^·d	IV/PO	bid	1–4	If WBC ≥ 50 × 10^9^/L, single dose (3 mg/m^2^) Dex was added on d0 and immature cells in peripheral blood were measured on d5.
	For the children who used glucocorticoids for ≥ 4 days within 1 week, the window period was shortened according to the specific conditions. However, at least 2 days of full dose of glucocorticoids treatment was completed before the application of L-Asp. The time of the start of remission-induction chemotherapy was still recorded as d5.						
Remission induction	Pred	L/I/H	45 mg/m^2^·d*	PO	tid	5–28	Dose reduced on d 29-35. *T-ALL: Pred 60 mg/m^2^·d
	VCR		1.5 mg/m^2^·d	IV	qw	5, 12, 19, and 26	The highest dose was 2.0 mg
	DNR		25 mg/m^2^·d	IVgtt	qw	5 and 12	LR: if WBC <1.0 × 10^9^/L or ANC <0.3 × 10^9^/L on d12, the second dose was delayed; if WBC <1.0 × 10^9^/L or ANC <0.3 × 10^9^/L 7 days later and no immature cells were found in peripheral blood, the second dose was not administered.
	L-Asp	L	6,000 U/m^2^	IM/IV	qod	6–24 (10 times in total)	If MRD > 1% on d19, then single dose of Peg-Asp (2,000 U/m^2^) was administered instead. Each dose of Peg-Asp (2,000 U/m^2^) could be replaced with 10,000 U/m^2^ of Er-winia Asp or 6,000 U/m^2^ of E-coli Asp (3 times a week × 6 times).
	Peg-Asp	I/H	2,000 U/m^2^	IM		6 and 26	Each dose of Peg-Asp (2,000 U/m^2^) could be replaced with 10,000 U/m^2^ of Er-winia Asp or 6,000 U/m^2^ of E-coli Asp (3 times a week × 6 times).
	IT^@^	L				5 and 19	For children with CNS-2 or got injury on the first bone marrow aspiration, additional doses on d8, d12, and d15 were added.
		I				5, 12, and 19	For children with T-ALL, CNS-2, CNS-3, or injury on the first bone marrow aspiration, additional doses were added on d8 and d15.
		H				5, 8, 12, 15, and 19	When the interval of IT administration was <1 week, the renal functions and MTX concentration were monitored, and folinic acid (5 mg/m^2^, q6h) was added for rescuer when necessary.
	CTX	L/I/H	1,000 mg/m^2^	IVgtt		29	For the children with WBC > 4.0 × 10^9^/L and ANC > 1.0 × 10^9^/L, the drugs were administered on d27. For the children with WBC <2.0 × 10^9^/L or ANC <0.8 × 10^9^/L, the drugs were administered on d33. The doses of 6-MP and Ara-C were reduced by half if WBC <2.0 × 10^9^/L or ANC <0.8 × 10^9^/L on d33.
	Ara-C		50 mg/m^2^	IH	q12h	29–35	
	6-MP		60 mg/m^2^·d	po	qn	29–35	
	IT					29	
	VCR	T-ALL, and the ones with MRD ≥ 1% on d19	1.5 mg/m^2^	IV	qw	50 and 57	If WBC <2.0 × 10^9^/L, ANC <0.8 × 10^9^/L, or PLT <80 × 10^9^/L at least 2 weeks after the completion of the last CAT, the therapy was delayed for 1 week. The doses of 6-MP and Ara-C were reduced by half if WBC <2.0 × 10^9^/L, ANC <0.8 × 10^9^/L, or PLT <80 × 10^9^/L 1 week later. Each dose of Peg-Asp (2,000 U/m^2^) was replaced with 10,000 U/m^2^ of Er-winia Asp or 6,000 U/m^2^ of E-coli Asp (3 times a week × 6 times).
	Peg-Asp		2,000 U/m^2^	IM/IV		50	
	CTX		1,000 mg/m^2^	IVgtt		50	
	Ara-C		50 mg/m^2^	IH	q12	50–56	
	6-MP		60 mg/m^2^·d	po	qn	50–56	
	IT					50	

This study was approved by the Ethics Committee of Guangzhou Women and Children's Medical Center. All parents signed informed consent before study initiation.

During the remission-induction chemotherapy, we compared the height, weight, waist, and hip circumference at discharge vs. at diagnosis. In addition, we measured Leptin and Adiponectin levels on days 0, 19, and 46 together with MRD using bone marrow examinations. A paired *t*-test was performed to evaluate data between days 0, 19, and 46.

### Risk Classification

Risk classification was based on the CCCG-ALL-2015 protocol which was modified from the St. Jude total XV protocol (14) after considerations specific to the Chinese population (18). Risk classifications were based on the following characteristics (age, white blood cell count, immunophenotype, and cytogenetics) and treatment response as measured by MRD levels during remission induction (on day 19) and at the end of remission induction therapy (on or around day 46).

### Remission-Induction Chemotherapy

Remission-induction chemotherapy was the most important and initial portion of the entire ALL chemotherapy regiment. Intravenous injection or oral administration of dexamethasone (Dex) (3 mg/m^2^, twice per day) was on days 1–4. Afterward, prednisone (15 mg/m^2^, three times per day) was orally administered on days 5–28. The prednisone dose was gradually reduced by half on days 29–35 until withdrawal. This was the initial remission-induction chemotherapy and was termed VDLP therapy. The second remission-induction chemotherapy was CAT therapy and consisted of cyclophosphamide (CTX), cytarabine (Ara-C), and 6-mercaptopurine (6-MP) (based on the St. Jude total XV protocol) (14) (Table 1). Children were discharged after 35 days of remission-induction chemotherapy, and then re-hospitalized for subsequent chemotherapy after 2 weeks.

### Data Collection

A medical record abstraction was performed for each patient. Height and weight were measured before chemotherapy and after 46 ± 2 days. Raw BMI was then calculated. The waist and hip circumferences were measured for each course of remission-induction chemotherapy. The waist-to-hip ratio (WHR) was then calculated. In addition, Leptin and Adiponectin levels in the bone marrow were measured at days 1–4, 19 ± 2, and 46 ± 2 together with MRD using bone marrow examinations. The leptin-to-adiponectin ratio (LAR) was subsequently calculated. MRD was the main prognostic variable in this study.

For children between 1 and 3 years of age, Seca416 and Seca376 were used to measure the length and weight. For children > 3 years of age, Seca704s was used to measure height and weight. Waist and hip circumferences were measured using Seca201. One decimal was used for the results of height, waist circumference, and hip circumference, while two decimals were used for the results of weight. The measuring tools were regularly verified to ensure that they were accurate and easy to use and calibrated before use. All measurements were performed by two trained nurses according to the Standard Methods for Somatometry issued by the World Health Organization (WHO) in 2008^1^.

The RayBio^®^ Human Leptin enzyme-linked immunosorbent assay (ELISA) kit (RayBiotech, Inc., Georgia, America) and the RayBio^®^ Human Acrp30 ELISA kit (RayBiotech, Inc., Georgia, America) were used to measure the levels of leptin and adiponectin and were performed according to the manufacturer's instructions. Patient samples were diluted 30,000 times before measuring Adiponectin levels.

### Standardization and Definitions of BMI

Raw BMI values were converted to age and gender-adjusted *z-*scores using the Centers for Disease Control and Prevention's Year 2000 growth charts for patients 2–10 years (Epi Info 3.5)^2^. Length for weight z-scores was substituted for patients who had measured values prior to turning 24 months of age. Patients aged 2–10 years were defined as underweight, normal weight, overweight or obese according to whether their BMI was below the 5th percentile, in or above the 5th percentile but below the 85th percentile, in or above the 85th percentile but below the 95th percentile or in or above the 95th percentile, respectively (19). Percentage of measured/median was defined as a proportion of measured value in the median of the WHO reference.

### Primary and Secondary Outcomes Variables

The primary outcome was MRD and the change in BMI. Secondary outcomes included the changes in waist circumference, WHR, Leptin, and Adiponectin levels. MRD was determined at diagnosis, days 19 ± 2 and at days 46 ± 2 of remission-induction chemotherapy using a multiparameter 6-color flow cytometry performed on bone marrow samples with a sensitivity of 0.01% (20).

For bone marrow examinations, routine time points for bone marrow aspiration in clinical practices (days 0, 19, and 46) were selected. Patients were placed in the lateral position, and then bone marrow aspiration was performed from the posterior superior iliac spine. After obtaining 0.5–1 ml of bone marrow aspirate, samples were centrifuged and the supernatants were stored at −80°C until required (13). All bone marrow aspirations were performed by experienced physicians.

### Statistical Analysis

All data were analyzed using SPSS 25.0 win64 software (IBM, NY, USA). Continuous data were examined for normal distribution using the Kolmogorov–Smirnov test. Continuous variables with normal distribution were presented as means ± standard division (SD) and were analyzed using an independent-sample *t*-test (intergroup comparisons) or paired *t*-test (pre and post-therapy) or using repeated measure ANOVA and SNK *post-hoc* test (intergroup comparisons in time). Frequencies (percentages) were used to present categorical variables, and the chi-square test was used for intergroup comparisons. *P* < 0.05 was considered statistically significant, i.e., BMI at diagnosis was analyzed in four (underweight, normal, overweight and obese) subgroups. BMI percentile changes from diagnosis to the end of induction were analyzed as a continuous variable. MRD on day 19 and at the end of induction were compared across the groups by the chi-square tests. Complete remission was defined as having <5% leukemic blasts with restoration of normal hematopoiesis.

## Results

### Patient Recruitment and Baseline Characteristics

In this study, 103 children with B-cell acute lymphoblastic leukemia (B-ALL) were enrolled. Six children were excluded due to severe disease needing intensive care, and three due to history of pancreatitis. Another 12 children were withdrawn during the study, which included five who were administered parenteral nutrition after refusing oral diet or enteral nutrition due to severe oral ulcers and seven who had variable blood glucose levels and received a diabetic diet. Finally, 82 children were included in the study. Baseline characteristics including age, gender, admission age, and risk classification by BMI category are presented in Table 2, while discharge status is presented in Table 3.

**Table 2:** Patient characteristics based on body mass index.

**Patient characteristics**	**Total (*n* = 82)**	**BMI subgroups**				***P*-value**
		**BMI < P5**	**BMI P5–P85**	**BMI P85–P95**	**BMI ≥P95**	
		**11 (13.4)**	**58 (70.7)**	**4 (4.9)**	**9 (11.0)**	
Age at diagnosis (years)	4.45 ± 2.44	6.23 ± 2.71	4.43 ± 1.95	3.87 ± 2.22	4.00 ± 3.21	0.179
**Gender**, ***n*** **(%)**						0.496
Male	38 (46.3)	6 (54.6)	24 (41.4)	2 (50.0)	6 (66.7)	
Female	44 (53.7)	5 (45.4)	34 (58.6)	2 (50.0)	3 (33.3)	
**WBC**, ***n*** **(%)**						0.895
<50 × 10^9^/L	61 (74.4)	8 (81.8)	45 (77.6)	2 (50.0)	6 (66.7)	
50–100 × 10^9^/L	12 (14.6)	2 (9.1)	7 (12.1)	1 (25.0)	2 (22.2)	
≥100 × 10^9^/L	9 (11.0)	1 (9.1)	6 (10.3)	1 (25.0)	1 (11.1)	
**Immunophenotype**, ***n*** **(%)**						0.929
B cells	68 (82.9)	9 (81.8)	49 (84.5)	3 (75.0)	7 (77.8)	
T cells	14 (17.1)	2 (18.2)	9 (15.5)	1 (25.0)	2 (22.2)	
**Total XV risk**, ***n*** **(%)**						0.186
Low	30 (36.6)	4 (36.3)	23 (40.0)	1 (25)	2 (22.2)	
Medium	43 (52.4)	5 (27.8)	31 (53.4)	1 (25)	6 (66.7)	
High	9 (11.0)	2 (18.2)	4 (6.9)	2 (50)	1 (11.1)	

*BMI, body mass index; WBC, white blood cells*.

**Table 3:** Patient physical characteristics at diagnosis and discharge.

	**Admission**	**Discharge**	**Paired *T*-test**	***P*-value**
BMI	15.65 ± 2.02	15.15 ± 1.57	2.515	**0.016**
BMI percentile	43.86 ± 27.98	43.75 ± 33.02	0.02	0.984
BMI *Z*-score	−0.14 ± 1.19	−0.46 ± 1.09	1.149	0.273
WFL percentile	39.81 ± 26.82	51.99 ± 32.05	−0.878	0.409
WFL *Z-*score	−0.19 ± 1.21	0.36 ± 1.74	−0.468	0.656
Waist circumference (cm)	51.81 ± 7.38	51.90 ± 7.38	−1.101	0.29
Abdominal circumference (cm)	54.44 ± 6.87	54.60 ± 7.57	−1.169	0.262
Hip circumference (cm)	53.44 ± 10.81	53.63 ± 11.33	−1.234	0.238
WHR	0.98 ± 0.06	0.99 ± 0.06	−0.683	0.506

*BMI, body mass index; WFL, weight for length; WHR, waist-to-hip ratio. P <0.05 are shown in bold*.

Minimal residual disease by BMI category is shown in Table 4. Among the four subgroups, abnormal BMI was associated with higher percentage of an MRD level of 1% or higher on day 19 (*P* = 0.04) and 0.01% or more on day 46 (*P* = 0.008). Percentage of an MRD level of 1% or higher on day 19 and 0.01% or more on day 46 was significantly higher in the higher BMI subgroups (overweight and obese) than the normal weight subgroup (7.7% vs. 0 and 30.8 vs. 6.9%, respectively).

**Table 4:** Patient minimal residual disease and adipocytokine levels based on body mass index.

**Patient characteristics**	**Total (*n* = 82)**	**BMI subgroups**				***P*-value**
		**BMI < P5**	**BMI P5–P85**	**BMI P85–P95**	**BMI ≥P95**	
		**11 (13.4)**	**58 (70.7)**	**4 (4.9)**	**9 (11.0)**	
**MRD on day 19**, ***n*** **(%)**
<0.01%	53 (64.6)	5 (45.5)	41 (70.7)	3 (75)	4 (44.4)	**0.04**
≥0.01 to <0.1%	15 (18.3)	2 (18.2)	10 (17.2)	0 (0)	3 (33.3)	
≥0.1% to <1	7 (8.5)	1 (9.1)	5 (8.6)	0 (0)	1 (11.1)	
≥1	3 (3.7)	2 (18.2)	0 (0)	0 (0)	1 (11.1)	
No data	4 (4.9)	1 (9.1)	2 (3.5)	1 (25)	0 (0)	
**MRD on day 46**, ***n*** **(%)**
<0.01%	65 (79.3)	5 (45.5)	52 (89.7)	3 (75)	5 (55.6)	**0.008**
≥0.01% to <0.1%	8 (9.8)	2 (18.2)	4 (6.9)	0 (0)	2 (22.2)	
≥0.1% to <1	4 (4.9)	2 (18.2)	0 (0)	1 (25)	1 (11.1)	
≥1	0 (0)	0 (0)	0 (0)	0 (0)	0 (0)	
No data	5 (6.0)	2 (18.2)	2 (3.5)	0 (0)	1 (11.1)	
Leptin levels on day 0	60.19 ± 51.00	63.65 ± 58.45	54.65 ± 47.84	139.33 ± 65.16	85.02 ± 39.00	0.118
Leptin levels on day 19	156.55 ± 116.09	221.37 ± 120.58	146.22 ± 108.90	400.34 ± 195.49	65.31 ± 56.55	0.061
Leptin levels on day 46	125.25 ± 111.55	91.78 ± 47.15	125.58 ± 109.35	312.45 ± 153.63	104.22 ± 62.40	0.361
Adiponectin levels on day 0	1,499.94 ± 1,304.77	887.99 ± 626.09	1,595.10 ± 1,364.26	2,096.80 ± 1,847.29	1,046.56 ± 479.39	0.501
Adiponectin levels on day 19	1,746.82 ± 932.94	1,965.09 ± 1,623.12	1,788.11 ± 857.34	535.65 ± 265.38	1,296.60 ± 239.52	0.505
Adiponectin levels on day 46	1,501.10 ± 1,031.11	491.02 ± 184.96	1,573.21 ± 1,079.82	1,752.84 ± 937.54	2,018.86 ± 322.62	0.187
LAR on day 0	0.0660 ± 0.0901	0.0757 ± 0.0522	0.0552 ± 0.0798	0.2245 ± 0.2824	0.1003 ± 0.0832	0.062
LAR on day 19	0.1150 ± 0.1368	0.1296 ± 0.0369	0.0963 ± 0.0927	0.0746 ± 0.0694	0.0504 ± 0.0437	0.069
LAR on day 46	0.1192 ± 0.1655	0.2156 ± 0.3427	0.111 ± 0.1442	0.1783 ± 0.1485	0.0522 ± 0.0327	0.58

*BMI, body mass index; MRD, minimal residual disease; LAR, leptin-to-adiponectin ratio. P <0.05 are shown in bold*.

**Figure 1:**
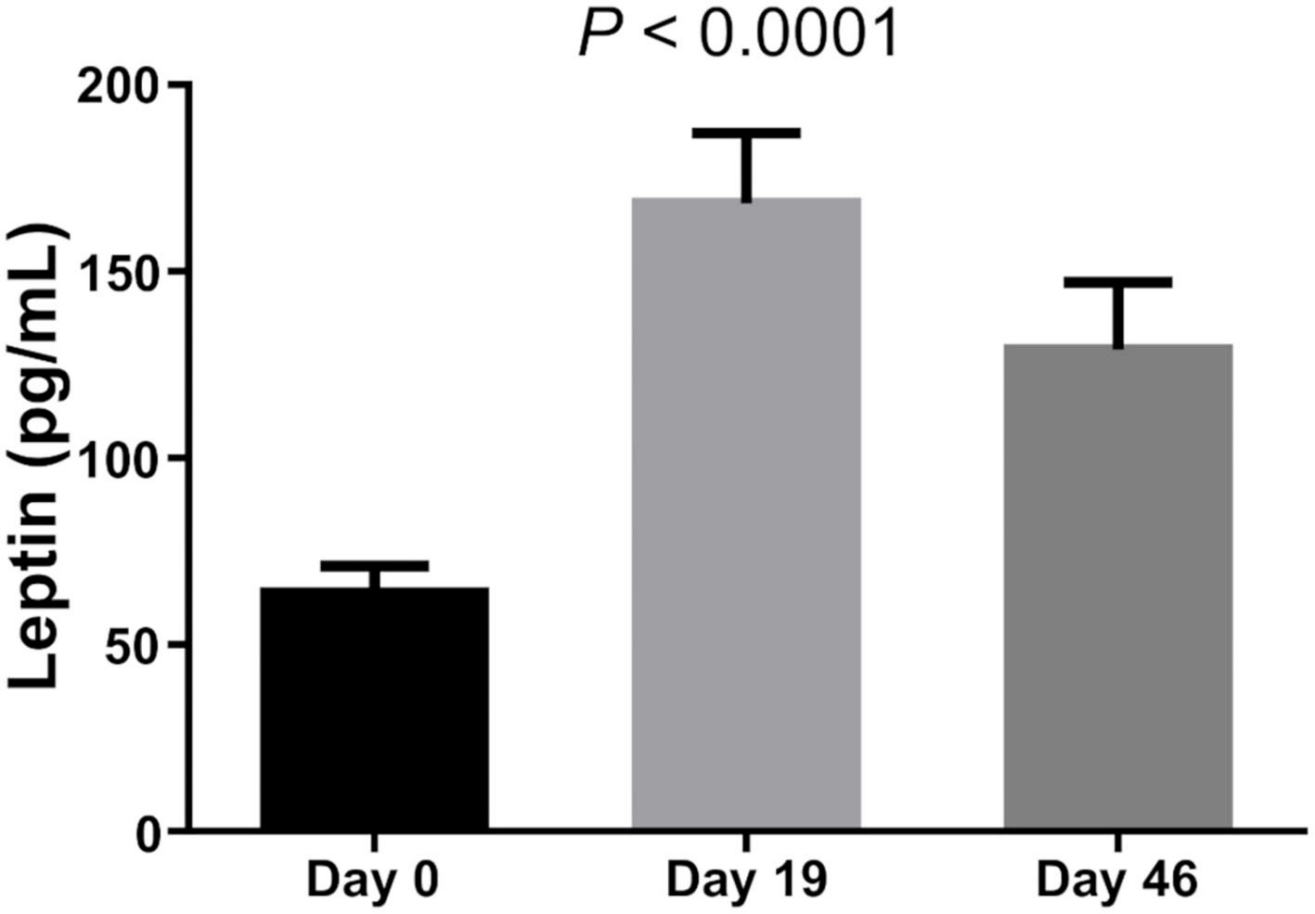
Leptin level of bone marrow.

**Figure 2:**
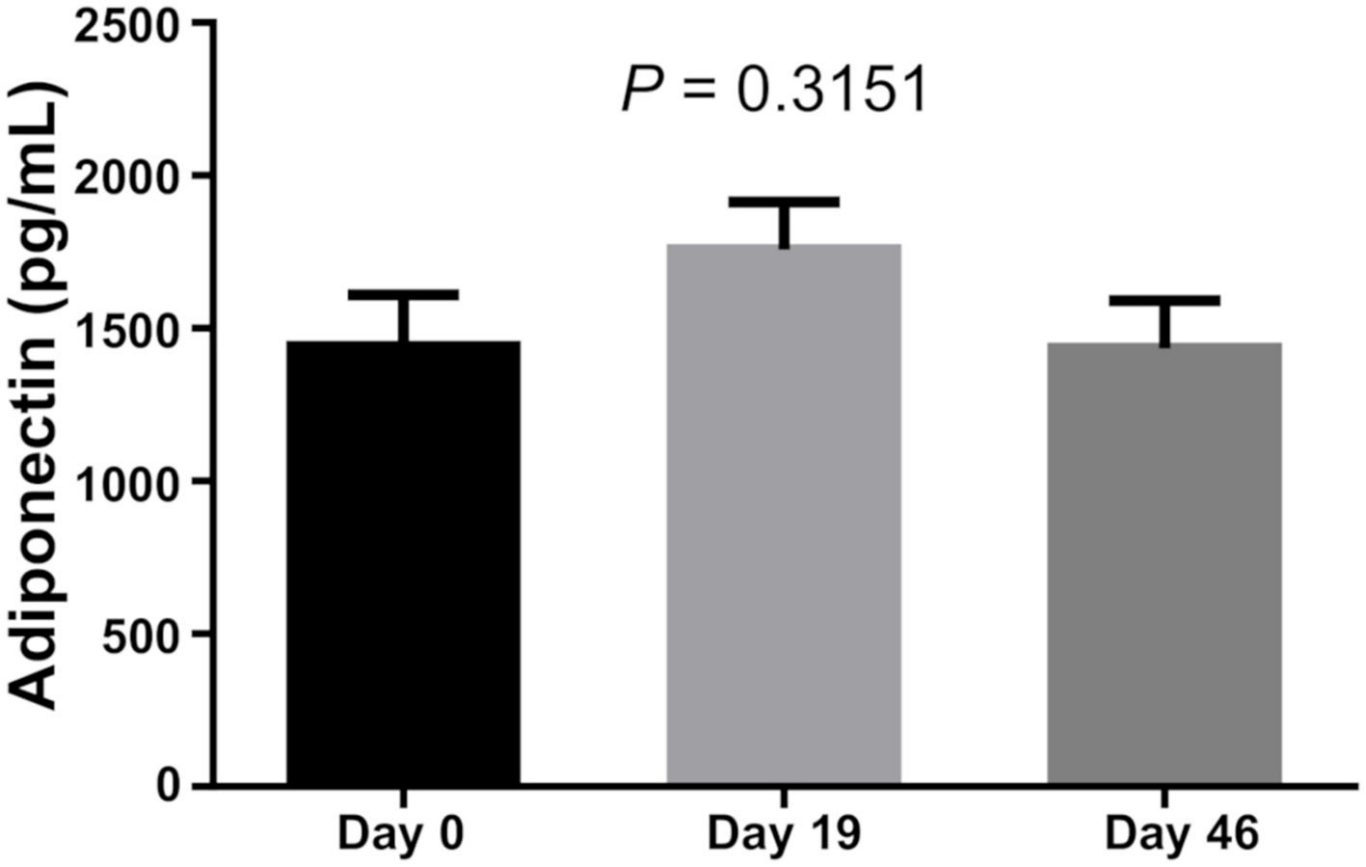
Adiponectin level of bone marrow.

**Figure 3:**
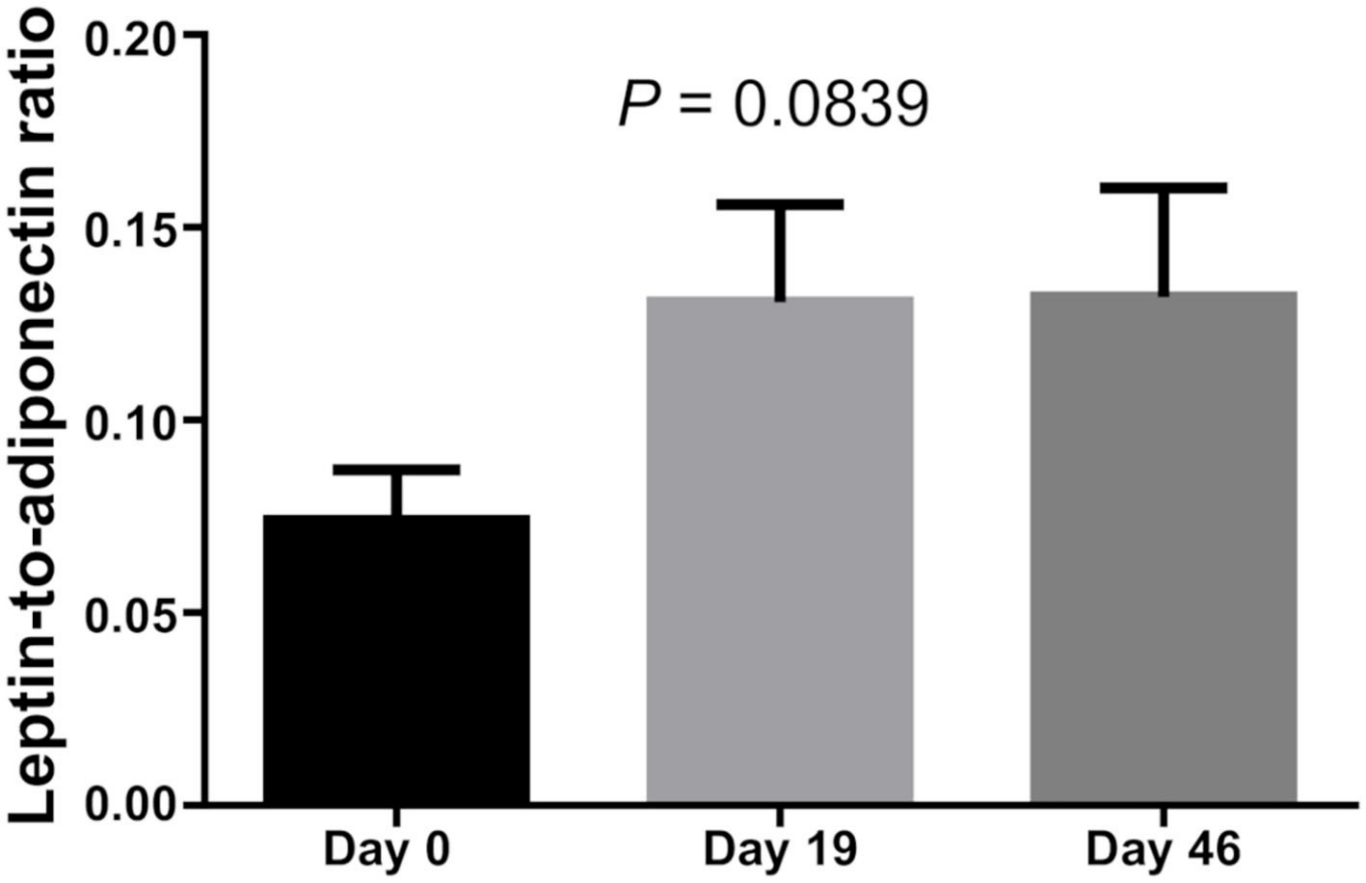
Leptin/Adiponectin rate of bone marrow.

There was no difference in Leptin and Adiponectin levels between BMI categories. However, paired *t*-test showed that both Leptin and Adiponectin levels raised significantly on day 19 of induction (*P* = 0 and *P* = 0.015, respectively; Table 5; Figures 1–3).

**Table 5:** Comparison of Leptin and Adiponectin marrow levels at different times.

	**Paired *T*-test**	***T*-value**	***P*-value**
Pair 1	Lep1 vs. Lep2	−5.3854	**0**
Pair 2	Lep2 vs. Lep3	2.086	**0.047**
Pair 3	Lep1 vs. Lep3	−2.6397	**0.013**
Pair 4	Adip1 vs. Adip2	−2.5552	**0.015**
Pair 5	Adip2 vs. Adip3	1.8865	0.07
Pair 6	Adip1 vs. Adip3	−0.7359	0.468
Pair 7	LAR1 vs. LAR 2	−2.5384	**0.016**
Pair 8	LAR 2 vs. LAR 3	0.0687	0.946
Pair 9	LAR 1 vs. LAR 3	−1.2887	0.208

*Lep1, Leptin levels at diagnosis; Lep2, Leptin levels at days 19 ± 2; Lep3, Leptin levels at days 46 ± 2; Adip1, Adiponectin levels at diagnosis; Adip2, Adiponectin levels at days 19 ± 2; Adip3, Adiponectin levels at days 46 ± 2. P <0.05 are shown in bold*.

The percentage of BMI at day 0/median was defined as a proportion of BMI at diagnosis within the median of the WHO reference and represents the difference between the measured value and the mean level for the same gender and age. The greater the difference between the BMI and reference value, the worse the MRD at day 46 (*P* = 0.014; Table 6).

**Table 6:** Pearson correlation analysis of body mass index, body fat distribution and minimal residual disease day 46.

	**Pearson correlation coefficient**	***P*-value**
BMI day 0	0.02	0.903
Percentage of BMI day 0/median	0.601*	**0.014**
Waist circumference day 0	0.623**	**0.01**
Waist circumference day 19	0.663**	**0.007**
Waist circumference day 46	−0.469	0.289
Hip circumference day 0	0.583*	**0.018**
Hip circumference day 19	0.597*	**0.019**
Hip circumference day 46	−0.352	0.439

BMI, body mass index. P <0.05 are shown in bold. ^*^P <0.05; ^**^P <0.01.

Waist and hip circumference reflected the distribution of body fat, i.e., the concentric distribution. The results suggested that fat concentric distribution is significantly correlated with poor MRD at day 46 (Table 6).

There was a significant positive correlation between BMI and bone marrow Leptin levels at diagnosis (*P* = 0.005) (Table 7), i.e., patients with higher BMI had higher levels of Leptin, suggesting Leptin resistance. No correlation was found between BMI and Adiponectin levels.

**Table 7:** Pearson correlation analysis of body mass index and adipocytokine levels.

		**Lep1**	**Lep2**	**Lep3**	**Adip1**	**Adip2**	**Adip3**
BMI day 0	Pearson correlation coefficient	0.535**	0.34	0.007	−0.091	−0.119	−0.164
	*P*-value	**0.005**	0.198	0.978	0.65	0.673	0.515
**Percentage of BMI-day 0 to medium**	Pearson correlation coefficient	0.451*	−0.054	−0.159	−0.539	−0.245	−0.175
	*P*-value	**0.031**	0.908	0.662	0.135	0.597	0.629

BMI, body mass index; Lep1, Leptin levels at diagnosis; Lep2, Leptin levels at days 19 ± 2; Lep3, Leptin levels at days 46 ± 2; Adip1, Adiponectin levels at diagnosis; Adip2, Adiponectin levels at days 19 ± 2; Adip3, Adiponectin levels at days 46 ± 2. P <0.05 are shown in bold. ^*^P <0.05; ^**^P <0.01.

## Discussion

Acute lymphoblastic leukemia is the most common pediatric cancer. Childhood survivors of acute lymphoblastic leukemia (SALL) are at risk of obesity and related cardiometabolic diseases, including type 2 diabetes, hypertension, stroke, and cardiovascular events (21).

We observed changes in BMI, waist circumference and MRD of the 82 enrolled ALL children during their remission-induction therapy (Table 1). In our study, 13 (15.6%) were overweight/obese and 11 (13.4%) were malnourished at diagnosis based on the age- and sex-adjusted BMI. Waist/hip circumference ratio in most children, i.e., 60 (73.2%) reached >1.0 on Day 19 during chemotherapy. Waist circumference increased in 22 children (27%) and hip circumference increased in 33 (40%) ALL children during remission-induction. A higher BMI was associated with significantly increased MRD at day 46 and showed a positive correlation (*P* = 0.014). In addition, we found positive correlations between weight, hip circumference, and MRD at day 46. This suggested that an increase in MRD was associated with concentric distribution of body fat. Our study suggested that monitoring and control of body weight during chemotherapy is important to the prognosis of the disease.

A meta-analysis suggested that a higher BMI at diagnosis was associated with poorer survival in children with pediatric ALL or acute myeloid leukemia (AML). Orgel et al. performed a meta-analysis of 4,690 cases of ALL and observed poorer event-free survival (EFS) in children with a higher BMI compared to children with lower BMI. A higher BMI was associated with significantly increased mortality and a statistically non-significant trend toward the greater risk of relapse compared to children with a lower BMI (22). At a time when obesity has become a global public concern, the use of hormone drugs such as dexamethasone administered during ALL treatment could cause obesity, especially in children from developing countries. Núñez-Enríquez et al. (9) performed a multicenter cohort study in a Mexican cohort of 1,070 children. Early mortality and early relapses were the main outcomes. Overweight/obesity at diagnosis were predictors of early mortality. In this study, the correlation between BMI at diagnosis and MRD at day 46 supports the results above. Importantly, the weight gain induced during remission-induction therapy (within 6 weeks after diagnosis) after glucocorticoid administration was further exacerbated after completion of therapy. This was regardless of the patients' clinical characteristics, such as age, race, gender, or treatment risk (23).

Body composition analysis also showed an increased incidence of skeletal muscle mass loss (24). Marriott CJC conducted body composition in 75 long-term survivors of ALL (more than 10 years after initial diagnosis). Excess fat mass and the combined morbidity of sarcopenic obesity were found to be prevalent in long-term survivors of ALL. This places ALL survivors at double jeopardy from excess body fat and inadequate skeletal muscle mass (SMM). This is associated with an adverse impact on overall survival (24). In our study, we found the correlation was not only between BMI at diagnosis and MRD day 46 but also between waist circumference and MRD at day 46. This finding suggests that body composition could also affect the outcome of ALL children.

Leptin is an effective adipocytokine to control obesity (12). We found that bone marrow leptin average levels as well as Adiponectin levels increased significantly during dexamethasone administration. Hypothalamic leptin resistance should be considered in obese patients with ALL (25). Obese leukemic survivors have been reported to have lower serum adiponectin levels compared to non-obese survivors (26), while dysregulation of the adipokine pathway has been associated with carcinogenesis and ALL (27). This study also confirmed that Leptin levels of those whose BMI was high at diagnosis were also increased, while two MS markers showed a positive correlation.

Minimal residual disease (MRD) is defined as the presence of sub-microscopic levels of leukemic cells (28). Detection of MRD during remission induction and consolidation therapy is the most sensitive method to evaluate treatment response and one of the strongest predictors of outcome in childhood acute lymphoblastic leukemia. Several studies have demonstrated the prognostic significance of measuring MRD in childhood ALL, suggesting that MRD positivity at serial time points during the treatment is highly predictive of relapse, and associated with poor treatment outcome (29, 30). A recent study suggested that MRD levels at the end of remission induction therapy measured using multiparameter flow cytometry have clinical significance in childhood ALL. High levels of MRD were strongly related to poor treatment outcomes (31, 32). This study found that the MRD day 46 was correlated with higher age- and sex-adjusted BMI at diagnosis (*P* = 0.014). MRD day 46 seemed worse in patients with fat concentric distribution, i.e., increased waist circumference on diagnosis, day 19 (*P* = 0.010 and *P* = 0.007). However, it did not seem to affect MRD through changes in adipocytokine levels.

There were several limitations to this study. Although we focused on the BMI of ALL children, the body composition measurements were not performed to access fat-free mass and fat distribution. In addition, longer longitudinal follow-ups were not performed on ALL children until the end of maintenance therapy and analysis for EFS and overall survival (OS) (33–36).

## Conclusions

In the study, we found that 13 (15.6%) ALL children were overweight/obese and 11 (13.4%) were malnourished (wasting) at diagnosis based on age- and sex-adjusted BMI. A higher BMI was associated with significantly increased MRD at day 46 and showed a positive correlation (*P* = 0.014). In addition, we found a positive correlation between weight, hip circumference at diagnosis, days 19, and MRD at days 46. Both BMI and fat concentric distribution affected the outcome of ALL children. A higher BMI was also associated with significantly increased Leptin levels at diagnosis. Leptin resistance should be a risk factor in ALL children with high BMI.

To address this risk of weight gain, early interventions such as regular weight and height monitoring and dietary assessment involving a multidisciplinary team of oncologists, nurses, dietitians, physical therapists, psychologists, and endocrinologists should be consulted to evaluate ALL children, preferably starting during remission -induction chemotherapy (19, 37).

## Original Details of Risk Classification

### Total Therapy Study XV for Newly Diagnosed

#### Patients With Acute Lymphoblastic Leukemia

Principal Investigator: Ching-Hon Pui, M.D.

Co-Principal Investigator: John T. Sandlund, M.D. Departments of Hematology/Oncology St. Jude Children's Research Hospital.

### Risk Classification

Patients were classified into one of three categories (low-, standard-, or high-risk) based on age at admission, leukocyte counts, presence or absence of central nervous system (CNS) 3 status or testicular leukemia, immunophenotype, cytogenetic and molecular diagnosis, DNA index, and early response to therapy. Hence, definitive risk assignment (for provisional low-risk cases based on presenting features) will be made after completion of remission induction therapy. The criteria and the estimated proportion of patients for each category (based on data from Total XIII study) are provided below.

#### Criteria for Low-Risk ALL (~40% of Patients)

- B-cell precursor ALL with DNA index ≥ 1.16, TEL-AML1 fusion gene, or age 1–9.9 years and presenting WBC <50 × 10^9^/L.
- Must not have (i) CNS 3 status (≥5 WBC/:L of cerebrospinal fluid with morphologically identifiable blasts or cranial nervepalsy), (ii) overt testicular leukemia (evidenced by ultrasonogram), (iii) adverse genetic features [*t*_(9;22)_ or BCR-ABL fusion gene; *t*_(1;19)_ with E2A-PBX1 fusion gene; rearranged mixed lineage leukemia gene (MLL); orhypodiploidy (<45 chromosomes)], or (iv) poor early response (≥1% lymphoblasts on day 19 or 26 of remission induction, ≥0.01% lymphoblasts by immunologic or molecular methods on remission date).

#### Criteria for Standard-Risk ALL (~50% of Patients)

∙ All cases of T-cell ALL and those with B-cell precursor ALL that do not meet the criteria for low-risk or high-risk ALL.

#### Criteria for High-Risk ALL (~10% of Patients)

- *t*_(9;22)_ or BCR-ABL fusion.
- Induction failure or >1% leukemic lymphoblasts in the bone marrow on remission date.
- >0.1% leukemic lymphoblasts in the bone marrow in week 7 of continuation treatment (i.e., before re-induction I, ~14 weeks post-remission induction).
- Re-emergence of leukemic lymphoblasts by minimal residual disease (MRD) (at any level) in patients previously MRD negative.
- Persistently detectable MRD at lower levels.

## Data Availability Statement

The raw data supporting the conclusions of this article will be made available by the authors, without undue reservation.

## Ethics Statement

The studies involving human participants were reviewed and approved by No. 2017102415 file granted by the Ethics Committee of Guangzhou Women and Children's Medical Center. Written informed consent to participate in this study was provided by the participants' legal guardian/next of kin.

## Author Contributions

JS: conceptualization and writing—original draft. HH, MX, and HL: data curation. HZ: formal analysis. HC and JS: methodology. JC, RZ, LZ, JT, and XW: resources. XW and HH: supervision. JS and JC: writing—review and editing. All authors contributed to the article and approved the submitted version.

## Funding

This study was funded by the Nestle Health Science Project (grant number 2013B021800030).

## Conflict of Interest

The authors declare that the research was conducted in the absence of any commercial or financial relationships that could be construed as a potential conflict of interest.

## Publisher's Note

All claims expressed in this article are solely those of the authors and do not necessarily represent those of their affiliated organizations, or those of the publisher, the editors and the reviewers. Any product that may be evaluated in this article, or claim that may be made by its manufacturer, is not guaranteed or endorsed by the publisher.

## Footnotes

^1^Available online at: https://www.who.int/tools/child-growth-standards.

^2^Available online at http://www.who.int/childgrowth/software.

## Abbreviations

BMI: body mass index
ALL: acute lymphoblastic leukemia
MRD: Minimal Residual Disease
MS: metabolic syndrome
HOMA-IR: homeostasis model of assessment for insulin resistance index
Dex: dexamethasone
CTX: cyclophosphamide
Ara-C: cytarabine
6-MP: 6-mercaptopurine
WHR: waist-to-hip ratio
LAR: leptin-to-adiponectin ratio
WHO: World Health Organization
ELISA: enzyme-linked immunosorbent assay
SD: standard division
B-ALL: B-cell acute lymphoblastic leukemia
SALL: survivors of acute lymphoblastic leukemia
AML: acute myeloid leukemia
EFS: event-free survival
SMM: skeletal muscle mass
OS: overall survival
CNS: central nervous system
MLL: mixed lineage leukemia gene.
